# Lived experiences of adolescents admitted for first-episode psychosis in South Africa

**DOI:** 10.4102/sajpsychiatry.v29i0.1960

**Published:** 2023-02-28

**Authors:** Luzuko Magula, Anusha Lachman, Rizwana Roomaney

**Affiliations:** 1 Department of Psychiatry, Faculty of Medicine and Health Sciences, Stellenbosch University, Cape Town, South Africa; 2 Department of Psychology, Faculty of Arts and Social Sciences, Stellenbosch University, Stellenbosch, South Africa

**Keywords:** adolescents, first episode psychosis, inpatient, cannabis, quality of care, South Africa

## Abstract

**Background:**

First-episode psychosis is common in adolescents and can be distressful to the person experiencing it for the first time. However, there is limited research globally and specifically in Africa about the lived experiences of adolescents admitted into a psychiatric facility for first-episode psychosis.

**Aim:**

To understand the adolescents’ experiences of psychosis and receiving treatment in a psychiatric facility.

**Setting:**

Adolescent Inpatient Psychiatric Unit, Tygerberg Hospital, Cape Town, South Africa.

**Methods:**

This was a qualitative study that used purposive sampling to recruit 15 adolescents with first-episode psychosis and admitted to the Adolescent Inpatient Psychiatric Unit, Tygerberg Hospital in Cape Town, South Africa. Individual interviews were audio recorded, transcribed and analysed using thematic analysis consisting of both inductive and deductive coding.

**Results:**

The participants described negative experiences of their first episode psychosis, provided varying explanations for their first episode psychosis and had the insight that cannabis precipitated their episodes. They described both positive and negative interactions with both the other patients and staff. They did not wish to return to the hospital again following their discharge. Participants stated that they wanted to change their lives, return to school and try to prevent a second episode of psychosis.

**Conclusion:**

This study provides insights into the lived experiences of adolescents presenting with first-episode psychosis and calls for future research to delve deeper into factors that support or enable recovery among adolescents with psychosis.

**Contribution:**

The findings of this study call for improving the quality of care in the management of first-episode psychosis in adolescents.

## Introduction

First-episode psychosis, a presentation of psychotic symptoms for the first time in a clinical setting by someone who has not previously been treated for psychosis before,^1^ is common in adolescence and can be disturbing, confusing and unfamiliar to the person experiencing it for the first time.^2^ The aetiology consists of a combination of biological, environmental and social causal factors.^3^ With a worldwide increase in mental health disorders among adolescents, it is important to understand the experiences of first episodes of psychosis in this population (Correll, 2018).^4^ Access to mental health care services for adolescents experiencing first-episode psychosis remains a challenge. In the Western Cape, South Africa, access to mental health services remains largely inadequate, with approximately 95% of adolescents in rural areas and 65% of adolescents in urban areas unable to access these services (personal communication). Stigma, poverty and discrimination against adolescents with mental health illnesses may be additional barriers to accessing services.^5^ Barriers to accessing care may be worsened by adolescents with behavioural disturbances who may be comorbidly abusing substances or have become violent and uncontrollable during their psychotic episodes.^6^ In settings where adolescents have limited social support, accessing care for mental illness may be more challenging. This is particularly critical in low- to middle-income settings, where psychosocial pressures on caregivers further disadvantage children from being identified and treated early for psychosis (personal communication). Community and family education and interventions may be necessary to bridge this gap of delayed and limited access to mental health services as they play an important role in adolescents seeking and receiving mental health support.^7^

In South Africa, adolescents experiencing psychosis who pose a risk to themselves, others and property are admitted to inpatient psychiatric units. These adolescents often express a need to leave immediately after admission even if they are still experiencing psychosis.^8^ Furthermore, patients transferred from other hospitals commonly verbalise the need to go home as they have already had long stays or an unpleasant experience in the referring hospital.^9^ On average, the duration of stay for adolescents with psychotic disorders in a psychiatric unit is 37 days.^10^

Few studies have described the experiences of adolescents following a first episode of psychosis. A study conducted in New Zealand explored the experiences of 14 adolescent participants admitted for first-episode psychosis.^11^ The adolescents described a range of experiences including changes in behaviour and interests. They also described having changes in mood, unusual physical sensations, hallucinations, delusions and challenges with their thinking processes. The adolescents felt that the psychosis placed them in danger of exploitation or harm to themselves. They attributed their psychosis to illegal drugs, physical illnesses and spiritual experiences. Furthermore, participants described positive experiences related to the staff and service accessibility, supportive and sensitive staff, an adequate continuation of care and peer support within the services. Others describe negative experiences including being hospitalised and being exposed to peers that were unwell and insensitive staff. Adolescents also commonly described increased isolation following an episode of psychosis and found this as a hurdle on their road to recovery.^12^ Some struggled with reintegrating back into their environments, including returning to school.^13^

Overall, there is limited research in Africa and across the world about the lived experiences of adolescents admitted into a psychiatric facility for first-episode psychosis. This article describes the lived experiences of adolescents admitted into a psychiatric unit in Cape Town, South Africa, for first-episode psychosis.

## Research methods and design

### Study design

A qualitative study was conducted consisting of semi-structured interviews between January 2020 and June 2021.

### Study setting

The study was conducted at the Adolescent Psychiatric Inpatient Unit at Tygerberg Hospital, Cape Town, South Africa. The unit provides inpatient services for adolescents between the ages of 13 and 18 years old. Patients with mental and related illnesses were referred to the unit from primary, secondary and tertiary level care, as well as from private practices. Tygerberg Hospital serves a predominantly low- to middle-income population that is exposed to community violence, illicit substance exposure, poverty, social adversity and high rates of unemployment.

### Study sample

Using purposive convenient sampling, participants were adolescents between the ages of 14 and 18 years inclusive who were admitted to the hospital for a first psychotic episode. A final sample size of 15 participants was determined adequate for this study as the data from these participants were rich and fully explored and answered the research question. The data were saturated as no additional information and perspectives were being identified.^14^ All participants were admitted as involuntary inpatients under the *Mental Health Care Act of South Africa* (*Act no 17 of 2002*).^15^ Participants were compensated with gender-neutral hygiene packs.

#### Inclusion criteria

The primary inclusion criterion was first-episode psychosis, defined in this study as a presentation of psychotic symptoms for the first time in a clinical setting by someone who has not previously been treated for psychosis before. This criterion was established using the Structured Clinical Interview for the Diagnostic and Statistical Manual of Mental Disorders, Fifth Edition (DSM5) (SCID-5).

#### Exclusion criteria

Participants who had two or more prior episodes of psychosis were excluded. Resolved first-episode psychosis cases before admission into the unit were excluded.

### Study procedure

The researchers did not form part of the multidisciplinary team (MDT) treating the patients and were not involved in any decision-making about the treatment course or the management and discharge of the patients from the ward. The primary researcher informed the MDT of the study, including the inclusion and exclusion criteria and the MDT referred patients meeting the criteria to the primary researcher.

Primary carers and their adolescents admitted to the ward were informed of the study by the researcher (verbally and study pamphlet) in their home language (Afrikaans, English, isiXhosa and isiZulu) and provided written informed consent and assent, respectively, if they agreed to participate in the study. Participants were informed that the primary researcher had no influence on their discharge from the unit, that their anonymity would be protected and that they could withdraw from the study at any time.

Interviews were held in a private consultation room in the ward by the primary researcher when the participant was no longer psychotic. The absence of psychosis was established by liaising with the MDT and further confirmed by the primary researcher, who is well versed in the diagnosis of psychotic disorders as a specialist psychiatrist. The primary researcher made use of the Positive and Negative Syndrome Scale (PANSS) to confirm the absence of psychosis before conducting the interview.

During the interview, participants were asked to describe the following: (1) their experience of their first episode of psychosis, (2) their experience of being taken from home to a primary facility of care, (3) their experience of the difficulties faced with getting to that facility and accessing care, (4) their symptoms during their illness and their perceptions of the symptoms, (5) their stay in the inpatient unit and (6) the factors that they thought made their experiences favourable or unfavourable from the moment they left home and sought help up to the day of the interview. The interviews were not time limited and proceeded for as long as the participants had experiences to describe, with the interviews lasting between 30 min and 60 min. The interviews took place two to four weeks before the participants were to be discharged from the unit. Participants who relapsed and regressed following the interview were to be referred to the unit MDT to optimise medication and offer further psychiatric and psychological interventions while still being inpatients. However, none of the participants relapsed because of or following the interviews and were discharged as scheduled. Reports of any form of abuse disclosed in the interviews were relayed to the MDT who would report to the Mental Health Review Board. The researchers did not form part of the MDT, and the power disparities between the researcher and the participant did not negatively impact the data collection process. The data was rich and authentic, and none of the participants had any limitations or reservations about describing their lived experiences.

### Data analysis

All interviews were audio recorded, transcribed and entered into the data management programme, ATLAS.ti Mac (version 8.4.4).^16^ A thematic analysis was conducted using the guidelines provided by Braun and Clarke.17 The researchers familiarised themselves with the data by conducting the interviews and transcriptions and reading and re-reading the transcriptions. Initial codes were then assigned using a combination of inductive and deductive coding. This was followed by grouping codes to create themes. The researchers reviewed, defined and named themes on an ongoing basis, and once they could not generate any new codes and themes, they determined that the data was saturated. To ensure the trustworthiness of the work, the research team detailed every aspect of the study from the beginning to the end, which may facilitate the transferability of this data to other contexts and settings with adolescent psychiatric inpatient units. The research team also worked closely together to ensure that the analysis process was continually reviewed. As a result of this process, the findings were themes of the raw data that described the exact subjective experiences of the participants, and they answered the main research question. The interpretation of findings was not negatively impacted by the power disparities between the researcher and the participant.

### Ethical considerations

The study was approved by the Health Research Ethics Committee (HREC) of Stellenbosch University (S20/01/003). Permission to conduct this study was also sought from the National Department of Health (NDOH) and Management of Tygerberg Hospital. The study was conducted in accordance with South African Good Clinical Practice Guidelines^18^ and the Declaration of Helsinki.^19^ Pseudonyms were used when referring to participants.

## Findings

A summary of the participant’s demographic and clinical characteristics is summarised in Table 1. Participants had a mean (s.d.) age of 16.27 (1.35) years, 53.3% (*n* = 8) were male, and 86.7% (*n* = 13) attended school. Referrals were predominantly from district health facilities (*n* = 12, 80%). Participants were admitted for a mean (s.d.) of 2.73 (1.62) months at the inpatient unit.

**TABLE 1:** Participant demographic and medical characteristics (*n* = 15).

Characteristics	Mean	s.e.	*n*	%
**Age (years)**	**16.27**	**1.35**	**-**	**-**
**Gender**				
Female	-	-	7	46.6
Male	-	-	8	53.3
**Attending school**				
Yes	-	-	13	86.7
No	-	-	2	13.3
**Referral pathway**				
Direct admission	-	-	2	13.3
District	-	-	12	80.0
Tertiary	-	-	1	6.7
**Final diagnoses**				
Bipolar I disorder with psychotic features	-	-	7	46.7
Substance-induced psychotic disorder	-	-	4	26.7
Schizophrenia spectrum disorder	-	-	3	20.0
Major **depressive disorder** with psychotic features	-	-	1	6.7
**Treatment medication**				
Atypical antipsychotics	-	-	8	53.3
Mood stabilisers	-	-	6	40.0
Antidepressants	-	-	1	6.7
**Duration of admission** (months)	2.73	1.62	-	-

The participants were asked to describe the lived experience of their first episode of psychosis, and the following themes and subthemes were identified and discussed: early experiences of psychosis, the experience of receiving inpatient treatment and considerations related to leaving the hospital.

### Early experiences of psychosis

In describing their early experiences of psychosis, the participants described the following sub-themes: (1) their experiences of first episode psychosis, (2) explanations of their first episode psychosis and (3) insight into the illness and reasons for admission.

**Experiences of first episode psychosis:** All participants’ experiences of their first episode of psychosis were negative. Some participants reported that they feared for their lives and believed that people wanted to kill them. Some participants stated that they were confused throughout their experience. Others said that they knew that they were mentally unwell.

‘I was mentally disturbed, confused and not well, my mind was not my own. I felt unsafe. I felt like someone was going to kill me.’ (James, male, 17 years old, Grade 8 educational level)

A common description of the psychotic phenomena experienced was described as that of auditory and visual hallucinations:

‘The voices were telling me to get out of the house and walk to the road. They told me to just go and sometimes they just made me walk outside even if I didn’t know where I was going to. At night I would see knives and snakes.’ (Anne, female, 14 years old, Grade 6 education level)

**Explanation of first episode psychosis:** Many participants attributed their psychosis to supernatural phenomena. For example, some reported that they thought they were possessed by demons and that evil spirits were using them. Others believed that they were bewitched by family members because of certain family dynamics, with family members hating each other or being in conflict with one another:

‘It was almost something like a demon came into me, like the devil was using me. The evil spirits were too much for me that’s why I got sick.’ (Kajal, female, 17 years old, Grade 11 education level)

**Insight into the illness and reasons for admission:** Although most participants associated their psychosis with supernatural phenomena, their insight into reasons for admission to the hospital was not attributed to this. Instead, participants identified smoking illicit substances as one of the reasons why they were admitted into the ward. The most common substance used by the participants was cannabis. Eight participants were admitted with a diagnosis of a cannabis-induced psychotic disorder. Cannabis was perceived negatively by many of the participants as they believed it damaged their brains, resulting in the development of bad habits, such as stealing.

### Experience of receiving inpatient treatment

Participants described varying accounts of their experiences relating to their stay in the ward. Some participants said that they did not like their stay in the unit as they experienced verbal, emotional and physical abuse from staff members and other patients in the ward. They felt that the nursing staff were rude towards them and also reported that the security staff treated them unfairly. They thought that they were being judged negatively and unfairly by staff members because of their past mistakes or actions during their psychotic episode. Participants reflected that because their behaviour during their psychotic episodes was disruptive, aggressive and violent, staff continued to look at them with this same negative view after their episodes, resulting in negative treatment from the staff:

‘…she’s (referring to one nursing staff) very strict we just ask for toilet and she just say, “Go away!”’ (Lela, female, 17 years old, Grade 11 education level)

However, other participants described positive experiences during their stay. They appreciated the commitment and genuine care of some of the staff towards them:

‘It was not bad at all doc, I was feeling safe with all the securities, I was eating very nice food, I was getting my tablets.’ (Mlungisi, male, 17 years old, Grade 11 education level)

Peers also played an important role in the hospitalisation experience. Some participants stated that they were relieved to find other patients of similar age as them when they arrived in the ward. This made their stay more favourable and made them feel at ease. They described positive interactions with peers and formed friendships. Sharing their experiences with peers in the unit made them feel less isolated in terms of their mental health experiences and improved their insight into their mental health:

‘In general we interacted fine, they told me that they were in the same process that I was going through because we are all patients, so the interaction was good.’ (Siphe, male, 17 years old, Grade 11 education level)

However, some participants reported that they did not interact well with other patients. They described that they initially had physical and verbal altercations with the other patients about unwanted treatment, verbalisations and disruptive behaviour by the other, especially during their first week of admission to the ward. Some participants described other patients as rude and uncontained, negatively impacting their stay. This was distressing for participants:

‘…people I slept with they make me mad, really mad, and they make me sick. Also, they try to kill themselves in front of me while I’m sick.’ (Erik, 15 years old female, Grade 9 education level)

### Considerations related to leaving the hospital

The participants described varying inputs related to leaving the hospital, which included the following sub-themes: (1) life trajectory after discharge, (2) relapse and prevention of future psychotic episodes and (3) returning to the hospital.

**Life trajectory after discharge:** Participants were optimistic about their lives after being discharged from the hospital. All reported that they had set goals for themselves. Most planned to change their behaviour from what they displayed before admission, as they recognised their behaviour to be unacceptable. Some had noted that their behaviours before admission were rebellious and disrespectful towards adults and wanted to change this behaviour to be more acceptable. They also planned to assist in their households by firstly looking after their mental health, and secondly, taking responsibility for it. Furthermore, they indicated that they would help with chores and assist their parents, grandparents and younger siblings where they could:

‘I told myself I want to start a new life. I must change my life and stop being rebellious because I had become a very rebellious child at home who was not listening at all and disrespecting my mother. I will become a child once more and behave like a child.’ (Pozisa, female, 18 years old, Grade 9 education level)

Participants reported that they had plans to stop using substances as they had identified the substances as precipitators and perpetuators of their psychotic episodes and behavioural disturbances. They also noted bad friendships to be triggers for them engaging in unwanted behaviours and planned to change these as well:

‘When I’m getting out of the hospital I will never try to smoke that stuff [*cannabis*] anymore because it’s doing other stuff to your body and brain as well.’ (Kajal, female, 17 years old, Grade 11 education level)

‘The best thing to do to avoid the psychosis from happening is to build better relationships with my family and get better friends.’ (SP, male, 17 years old, Grade 8 educational level)

The participants were excited to return to school as they believed they needed education to successfully navigate the employment sector. They believed education was important to their growth and development and they wanted to make their parents proud:

‘I have to study for a test that’s coming in November. I’m hoping to get into university for Sociology.’ (Reba, 18 years, female, improving Grade 12 results)

**Relapse and prevention of future psychotic episodes:** Most of the participants were worried about getting ill again, as they did not want to return to the hospital. They were committed to preventing future psychotic episodes and endeavoured to improve their mental states. They identified taking their medication and following up as outpatients as necessary after discharge as this would be key to remaining in remission following their psychotic episode and would help prevent returning to the hospital:

‘I’m going stay in church, I’m going to do my things and duties that I need to do, I can’t drink, I can’t smoke cannabis, I can’t do anything I can only smoke cigarettes. I must drink my treatment and continue with my treatment.’ (Ruth,14 years old, female, Grade 6 educational level)

**Return to hospital:** Most of the participants did not want to return to the hospital for treatment. Some indicated that the hospital was far from their homes and their families were unable to visit them. Others would seek medical assistance at hospitals that would not admit them for long periods. Some stated that they would not return because of the bad experiences they had at the hospital including harsh and unfair treatments and pressures they were subjected to:

‘No, I don’t want to come back here because I like washing in the evening and here in the morning you must wake up 5 o’clock then you must wash for 5 minutes.’ (Leah, 17 years, female, Grade 11 education level)

## Discussion

Most studies have focused on the experiences and support needs of parents and family members of patients with first-episode psychosis^20^ or practitioner-centred views of working with patients with psychosis and their families.^21^ This study is one of a few that explored the lived experiences of adolescents admitted for first-episode psychosis into an adolescent inpatient psychiatric unit. The study produced three themes, namely early experiences of psychosis, the experience of receiving inpatient treatment and considerations related to leaving the hospital.

The study showed similar results as the study by Cadario^11^ with regard to the participants’ experiences of their first episode of psychosis, which included behavioural disturbances and psychotic symptoms including hallucinations and delusions. According to Hirschfeld et al.,^22^ most of their participants described vivid overwhelming, in-the-moment experiences of altered perception and sensation that impelled them to behave unusually. De Wet et al.^23^ also revealed that the participants described high levels of emotional distress relating to uncertainties at every stage of their illness, including concerns about their futures. The participants’ accounts in the study are consistent and show the need for a personalised understanding of each individual’s experience. Participants in the current study also had varying explanations for the causes and triggers of their psychotic episode. Some described the use of illicit substances like cannabis, while others described cultural and spiritual phenomena including being bewitched by family members or being possessed by demons. As described by Hirschfeld et al.,^22^ participants’ explanations covered a broad spectrum of ideas about internal and external factors influencing the onset of their psychosis. The clinical implication here is that multiple explanations may need to be embraced by mental health practitioners and that patients should be understood from within their own framework of the meaning of their first episode of psychosis.^24^ The insight of the participants into the reasons for their admission had markedly improved by the time of the interviews. Almost all participants knew that they had become mentally ill and had to be admitted. This was surprising as psychotic disorders are often associated with poor insight,^25^ with some even considering it a core symptom of psychosis.^26^ Although the relationship between insight and treatment adherence is complex and not clearly supported,^27^ most of the participants appreciated the need to adhere to treatment as recommended by the MDT to prevent relapse.

Participants were eager to change their behaviour and friendships after they were discharged. This may be because the relationships were already damaged during the episode of psychosis. As described in a recent study that explored the experiences of friendships of young people with first episode psychosis, withdrawal from friends because of mental illness and even the stigma from the friends when one is psychotic could be the reason the participants did not value their current friendships and wanted to change them.^28^ Contrary to the participants in the study by Huckle et al.,^27^ who saw a unique role for friends in their recovery and made conscious efforts to rebuild social networks, the participants of this study seemed to have an individualised and family-orientated approach to seeking support for their recovery and saw friends more as relapse triggers and illness perpetuators.

The participants had clear plans for their lives following their discharge. There was a longing to return to their families and change their behaviours. Participants stated that they wished to return to school or seek employment to support their families. However, these goals may need to be supported as research shows that patients find it difficult to reintegrate back into the community following admission for first-episode psychosis.^27^

This study outlines a way of understanding the participant’s subjective experiences of psychosis separate from those described by the referring or observing clinician or family. They also outline a unique observation to care different from that seen by the treating team or other staff. These lived experiences can be understood and interpreted using the biopsychosocial model of describing the illness and initiating care.^29^ As outlined by the biopsychosocial model of care, knowledge of these subjective lived experiences forms an essential contributor to their health outcomes and care. These findings also speak to the level of care that needs to be addressed, which does not focus on the biomedical model of care that includes medical treatment of documented symptoms only but holistically address concerns of humane treatment and care in the unit as well as in the community. For all the participants, the biopsychosocial approach in their management ensured that they had a positive experience of their inpatient treatment and overall experience of the first psychotic episode. These findings help to understand and appreciate the participant’s descriptions of their lived experience in a philosophical manner not purely governed by cellular and biological explanations of first-episode psychosis but by the biopsychosocial model that explains experiences of illness in all domains of human experiences.

Given the reported traumatic experiences of some adolescents in this study, an important consideration must be given to the initial presentation of adolescents to high secure units, with sensitivity given to the fear and distrust of the process of admission. This will in turn help minimise the risk if adolescents and the treatment team are aware of the precipitating factors that make the admission less traumatic for the adolescent.

The study had several limitations. The participants were admitted for different periods at different times. This could have impacted the experiences of the participants in terms of the staff allocated in the ward at the time of their admission. The coronavirus disease 2019 (COVID-19) pandemic and the lockdown regulations posed a challenge to the study as the levels of care, staffing and lived experiences would have differed between the participants interviewed before and during the pandemic. The participants were interviewed once, and this could have limited information provided as participants may have been under pressure or guarded in their first interview. The participants were not contacted again to validate the identified themes. Although recalling lived experiences during an episode of psychosis may be limited by recall biases and is a limitation, it is still very important to explore these experiences to get subjective and in-depth accounts of the lived experiences. The strength of the study was that the participants were interviewed as soon as they were not psychotic, and they could give an almost immediate account of experiences without recall biases.

## Conclusion

This study provides some insight into the lived experiences of adolescents presenting with first-episode psychosis. The study highlighted how these adolescents experienced their psychosis, their treatment in the facility and their hopes for the future. The findings provide insight into patients’ perceptions of their psychotic episodes and experiences within the hospital setting. Future research should delve deeper into factors that support or enable recovery among adolescents diagnosed with psychosis.
